# Aortosternal Venous Compression: A Review of Two Cases

**DOI:** 10.1155/2022/4591024

**Published:** 2022-10-05

**Authors:** Victoria Giglio, Zain Badar, Yasovineeth Bhogadi, Brian Van Adel, Gordon Yip

**Affiliations:** ^1^Department of Surgery, McMaster University, Hamilton, Ontario, Canada; ^2^Interventional Radiology Department, University of Toronto, Univeristy Health Network, Toronto, Ontario, Canada; ^3^Radiology Department, McMaster University, Hamilton, Ontario, Canada; ^4^Division of Neurology Neurosurgery and Diagnostic Imaging, Hamilton General Hospital, McMaster University, Hamilton, Ontario, Canada

## Abstract

Aortosternal venous compression (AVC) is a rare venous compression syndrome that involves brachiocephalic venous compression due to its positioning between the sternum and the aorta. One of the features of AVC involves compression of the left innominate vein with variability in luminal caliber on inspiration and expiration. Imaging modalities such as computed tomography (CT) examination can aid in initial diagnosis; however, venography can be utilized for confirmatory diagnosis due to its higher specificity during the inspiratory and expiratory phases. Through findings demonstrated during venography, we herein present two cases of confirmed AVC secondary to an aberrant right subclavian artery. Characteristic imaging features in the diagnosis of AVC and its etiology are discussed.

## 1. Introduction

Aortosternal venous compression (AVC) was first described by Wurtz et al. in 1989 [1]. It is a rare venous compression syndrome that is commonly asymptomatic in otherwise healthy patients due to compensation by collateral vasculature [2]. Scarcely reported symptoms in the literature include left upper extremity edema and intermittent instances of pain [1–3]. Although mediastinal masses can result in external compression of the underlying vasculature within the anterior or superior mediastinum, AVC is a diagnosis of exclusion. It can be caused by tortuous and/or ectatic great vessels resulting in associated compression on the underlying brachiocephalic vein in the setting of a diminished anterior-posterior chest diameter [2, 4, 5]. Three instances of an aberrant right subclavian artery have been reported in the literature as the causative agent of AVC; however, the prevalence of this etiology has yet to be established [2, 5].

We present two cases of AVC, in the setting of anatomical variation of aberrant right subclavian artery, wherein chest computed tomography (CT) and venography were utilized in establishing this rare diagnosis and its etiology.

## 2. Case Presentation

### 2.1. Case 1

A 72-year-old female presented with ongoing paresthesia and “pulsations” of the right neck and scalp. Her medical history includes an asymptomatic congenital coronary to pulmonary artery fistula that was monitored without any surgical intervention. The patient denied any loss of consciousness, tinnitus, or slurred speech. Physical examination demonstrated a lack of neck or facial swelling, lack of skin discoloration, and absence of dysphagia. A focused upper extremity exam revealed intact sensations, 2+ reflexes bilaterally, normal muscle tone and motor strength 5/5, in addition to a steady gait. Laboratory investigations showed an international normalized ratio (INR) of 1.0 and an activated partial thromboplastin time (aPTT) of 29. Complete blood count (CBC), Vitamin B12, and thyroid stimulating hormone (TSH) levels were all within normal limits.

**Figure 1:**
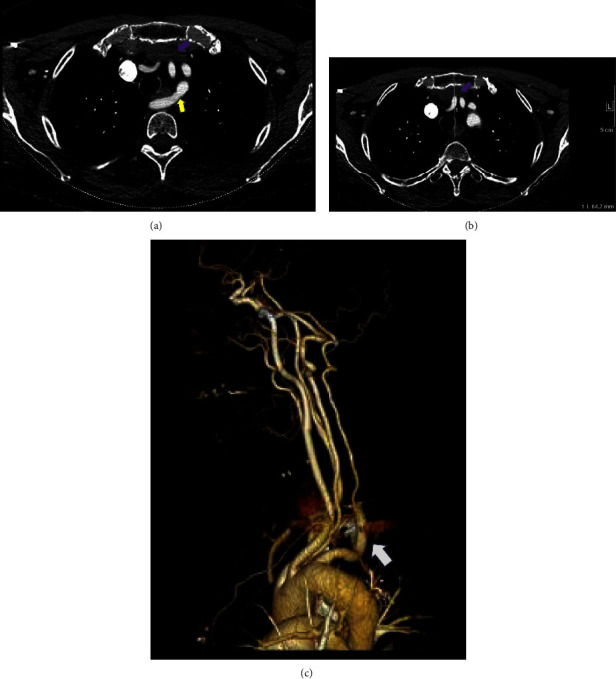
(a) Axial contrast-enhanced CT images (CTA neck) demonstrate the anatomical variation of an aberrant right subclavian artery (yellow arrow) with its course posterior to the esophagus, resulting in an impression along the dorsal aspect of the esophagus. The left brachiocephalic vein (purple arrow) demonstrates no evidence of stenosis, with the examination performed in the inspiratory phase. No evidence of diverticulum of Kommerell. Anteroposterior (AP) diameter of 8 cm on inspiratory phase. (b) Axial contrast-enhanced CT images (CTA neck): retrospective analysis demonstrates an anteroposterior (AP) diameter of 6.4 cm in the expiratory phase. (c) Volume rendered 3D reconstruction demonstrates an aberrant right subclavian artery (arrow) arising distal to the origin of the left subclavian artery and traverses the mediastinum posterior to the esophagus and the trachea.

**Figure 2:**
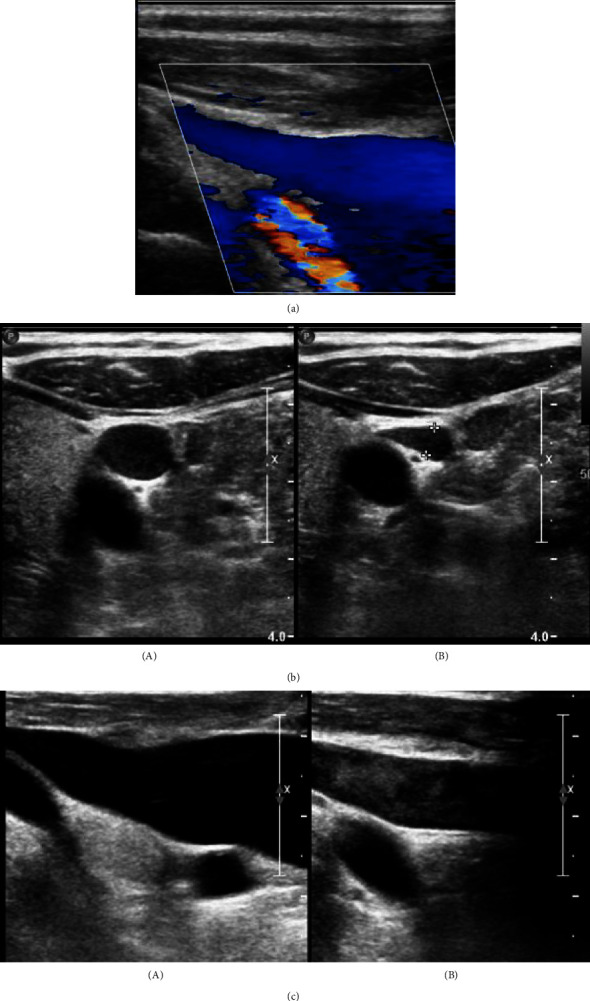
(a) Proximal segment of the left IJV demonstrates patency. (b) Diffuse slow flow of the compressible left IJV with the near stagnant flow in the proximal left IJV. (c) Comparison between the right IJV (A) and left IJV (B), with the left IJV demonstrating increased echogenicity of the blood suggesting underlying aggregation with near stagnant to and fro flow of blood. This layered appearance could be seen in the setting of underlying proximal compression/obstruction. Right IJV (A) was observed to be patent with the nonsluggish flow.

Due to the ongoing paresthesia, a contrast-enhanced CT head and neck was ordered. Initial imaging revealed an aberrant right subclavian artery with a course dorsal to the esophagus and an anteroposterior (AP) chest diameter of 6.4 cm (Figure 1). An ultrasound of the neck was ordered as part of the workup (Figure 2) which demonstrated no thrombus within either internal jugular vein (IJV) but did show the diffuse slow flow of the left IJV, with the near stagnant flow in the proximal left IJV. Findings were nonspecific; however, the layered appearance could be seen in the setting of underlying proximal compression/obstruction.

**Figure 3:**
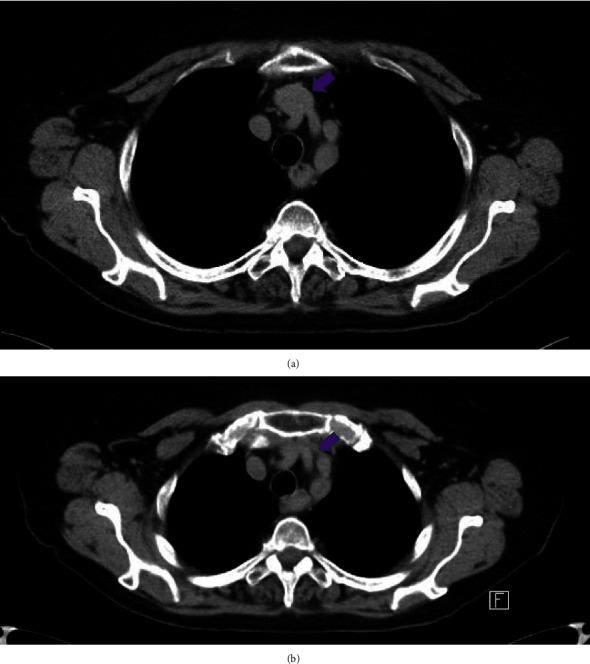
(a) Axial noncontrast CT obtained in full inspiration demonstrates patent left brachiocephalic vein (purple arrow). AP chest diameter: 8 cm. (b) Axial noncontrast CT obtained in full expiration demonstrates stenosis of the left brachiocephalic vein (purple arrow) as a result of compression between the left common carotid artery/left brachiocephalic artery and the manubrium. AP chest diameter: 5.6 cm.

**Figure 4:**
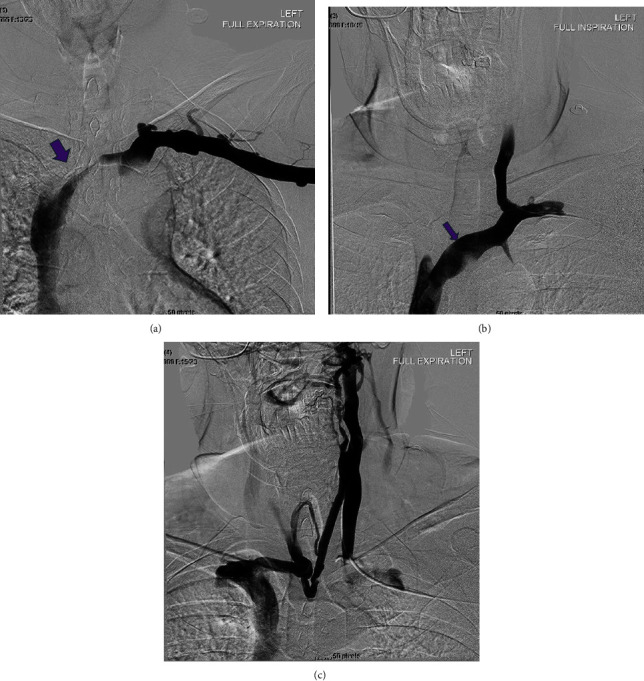
(a) Left basilic vein was cannulated and a venogram was performed (in expiration) via a 4F Omni flush catheter in the left axillary vein, which demonstrated severe stenosis of the left brachiocephalic vein (purple arrow). (b) Using a combination of KMP catheter and angled Glidewire, the left IJV was cannulated, with the KMP catheter subsequently exchanged for a 4F Omni flush catheter and a venogram was performed in inspiration demonstrating the prompt flow of contrast from the left IJV into the left brachiocephalic vein and subsequently into the SVC without significant stenosis. No filling defect to suggest thrombus. (c) Venogram within the left IJV (in full expiration) demonstrated contrast within the left IJV to become stagnant with multiple venous collaterals draining the left IJV via contralateral brachiocephalic and jugular veins.

The initial CT neck did not include the entire mediastinum within the field of view and thus additional imaging with a chest CT was ordered to rule out mediastinal mass as an etiology of proximal compression. The subsequent imaging did not reveal a mediastinal mass or aortic arch aneurysm, however, did demonstrate a left-sided aortic arch with an aberrant right subclavian artery without a diverticulum of Kommerell. However, as the CT chest examination was performed during the expiratory phase, findings of brachiocephalic venous compression were noted. A comparison was made to the prior CT angiography (CTA) neck, with interval difference in the brachiocephalic venous caliber. Thus, an inspiratory phase of the chest CT was also performed in the same setting to further evaluate the change in luminal caliber of the brachiocephalic vein. Findings of the chest CT (with inspiration and expiration) revealed the left brachiocephalic vein to become severely stenotic on expiration with an AP chest diameter of 5.6 cm. Comparatively, during inspiration, the chest CT revelated a patent left brachiocephalic vein with an AP chest diameter of 8 cm (Figure 3). Findings were suspicious for AVC, and thus venography was ordered. The venogram was obtained through a left upper extremity venous catheter during both expiration and inspiration. The venogram demonstrated severe stenosis of the left brachiocephalic vein during expiration with multiple venous collaterals around the site of obstruction (Figure 4). The venogram during inspiration demonstrated prompt flow of contrast across the left brachiocephalic vein with no evidence of obstruction. Previous exclusion of other aforementioned etiologies of venous compression, and the venographic findings mentioned above, led to a diagnosis of AVC in the setting of anatomical variation of an aberrant right subclavian artery.

Management by the neurology service included conservative options such as arm elevation at night. The patient opted out of having any surgical intervention. The patient has since been lost to follow-up.

### 2.2. Case 2

A 34-year-old female presented with a 6-month history of dysphagia and headaches accompanied by left orbital pain with visualization of occasional “squiggly lines” in the left eye. Additionally, she complained of a warm sensation overlying the left forehead and ipsilateral ear. She denied any pulsatile tinnitus, facial droop, or paresthesia. The medical history of the patient included tonsillectomy, with a family history of intracranial aneurysms, non-Hodgkin's lymphoma, colorectal cancer, and rheumatoid arthritis.

On physical examination, she was alert and oriented to person, time, and place. No cranial or carotid bruits were observed. Normal ocular vision with no pathological nystagmus, proptosis, or diplopia was noted. The facial sensation was intact, and the muscles of facial expression were normal and symmetric. Motor examination revealed normal bulk, tone, power, and coordination in all 4 limbs with no pathological reflexes. The sensation was grossly normal. Romberg's testing was negative. Physical examination demonstrated a prominent left frontal superficial scallop vein that became prominent when a patient was placed in a left lateral decubitus position. The left frontal superficial scallop vein did not demonstrate prominence during spine flexion, right lateral decubitus, or upright positioning.

Due to the ongoing headaches and the prominent left frontal superficial vein, investigation with MRI of the brain and MR angiography (MRA) was performed. Findings of the MRA demonstrated no evidence of dural arteriovenous fistula, vascular thrombosis, arteriovenous (AV) shunting, or vascular malformation.

**Figure 5:**
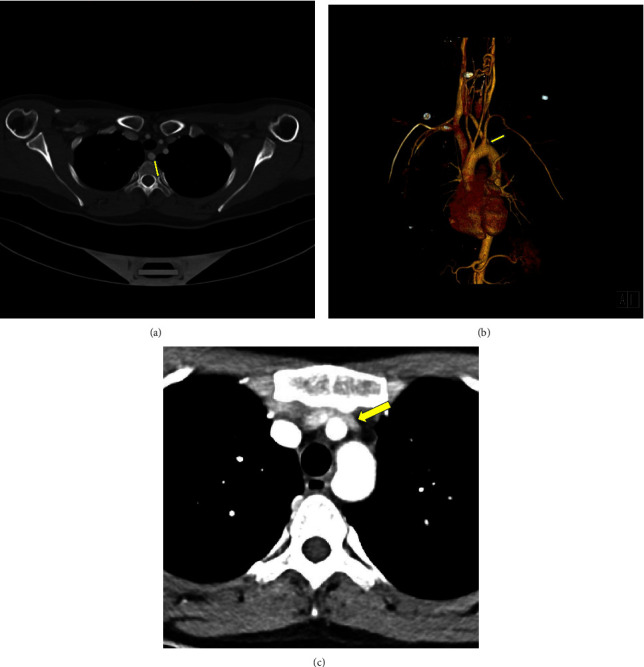
(a) Axial contrast-enhanced CT images (CTA neck) demonstrate the anatomical variation of an aberrant right subclavian artery (yellow arrow) with its course posterior to the esophagus. (b) Volume rendered 3D reconstruction demonstrates an aberrant right subclavian artery (arrow) arising distal to the origin of the left subclavian artery and traverses the mediastinum posterior to the esophagus. (c) Axial contrast-enhanced CT images (CTA chest) examination performed in the inspiratory phase demonstrates mild compression of the brachiocephalic vein (yellow arrow), however, patency is maintained. Anteroposterior (AP) diameter of 7.4 cm on inspiratory phase.

**Figure 6:**
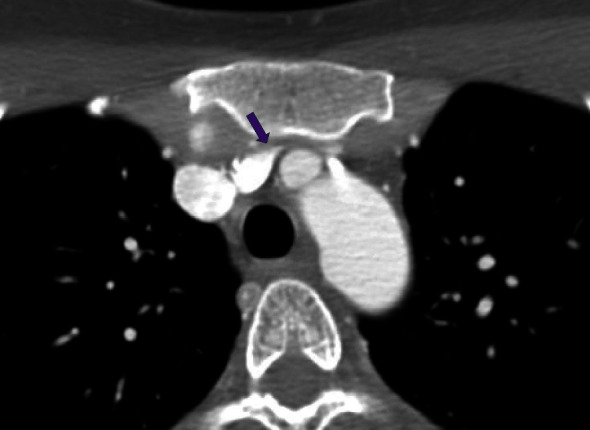
Axial contrast-enhanced CT images (CTA neck) (expiratory) demonstrate severe stenosis of the left brachiocephalic vein (purple arrow) with compression of the vein between the manubrium and the origin of the left common carotid artery. Anteroposterior (AP) diameter of 5.2 cm in the expiratory phase.

**Figure 7:**
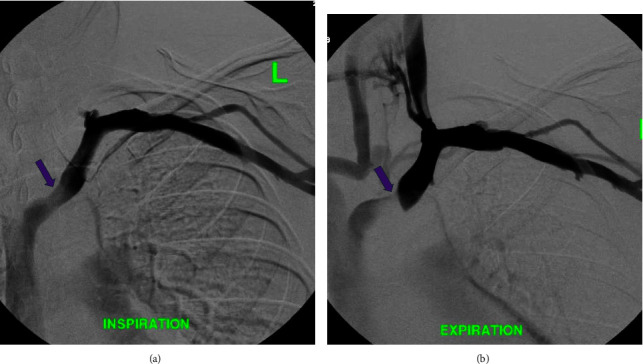
(a) With an intravenous cannula in the left antecubital fossa, the contrast was injected and a venogram was obtained in deep inspiration demonstrating patent left axillary, subclavian, brachiocephalic (purple arrow), and SVC. (b) Venogram (expiratory phase) demonstrates severe stenosis within the left brachiocephalic vein (purple arrow) with reflux of contrast within the left IJV. Collateral vasculature arising from the left IJV with communication to the right brachiocephalic is noted.

Further investigation with a CTA chest (inspiratory phase) was conducted to rule out a mediastinal mass resulting in venous compression. CTA chest demonstrated no evidence of a mediastinal mass; however, incidentally, an aberrant right subclavian artery was noted (Figure 5). Furthermore, there was mild compression of the brachiocephalic vein during this initial inspiratory phase, which warranted further investigation to rule out aortosternal venous compression (Figure 5(c)). CTA in an expiratory phase was subsequently performed, demonstrating findings of severe stenosis of the left brachiocephalic vein (Figure 6). Thus, a venogram was ordered as part of the workup in order to confirm the diagnosis of AVC. Findings of the venogram demonstrated a patent left subclavian and superior vena cava (SVC) with no significant stenosis with respect to the left brachiocephalic vein in the inspiratory phase (Figure 7(a)). The subsequent expiratory phase (Figure 7(b)) demonstrated severe stenosis of the left brachiocephalic vein, with cephalad reflux of contrast in the left IJV. Additionally, collateral supply arising from the left IJV and communicating with the right brachiocephalic vein was observed during the expiratory phase. The above findings were utilized to confirm the diagnosis of AVC with a component of cranial venous congestion.

The neurology team opted for conservative management including arm elevation at night. Based on the last follow-up visit the patient is doing well with decreased severity of symptoms.

## 3. Discussion

### 3.1. Symptoms and Presentation

Extrinsic venous compression can be observed in the setting of mediastinal mass compression given the rather confined anatomic space within the superior and anterior mediastinum [5, 6]. AVC, characteristically regarded as brachiocephalic vein compression, is a rare form of extrinsic venous compression, first reported in 1989 [1]. Tortuous and/or ectatic great vessels can act as another factor resulting in extrinsic compression of the brachiocephalic vein, particularly in patients with a diminished anteroposterior chest diameter. An aberrant right subclavian artery has also been associated with AVC [2, 4]. The prevalence of AVC has not been well characterized due to its rarity. A study in hemodialysis patients undergoing fistulograms identified some degree of extrinsic compression of the brachiocephalic vein in 44% of patients [7].

AVC is often asymptomatic, due to adequate compensation by collateral vasculature; however, cases in the literature have described instances of pain and swelling in the supraclavicular region and left upper extremity, as well as morning headaches [2, 8]. One case reported symptoms of paresthesia, thus, a similar symptomatic presentation to the first case presented in this case series. Even though rare, symptoms of paresthesia combined with pulsation, can lead the referring clinician to consider AVC as part of the differential workup [3]. Furthermore, the presence of an anatomical variant such as an aberrant right subclavian artery resulting in esophageal compression due to its dorsal course can result in dysphagia lusoria, which potentially accounts for the dysphagia observed in the second case presented above [2]. Nevertheless, the atypical presentation of AVC and the variable etiology of this syndrome highlight the necessity of combining imaging modalities in confirming a diagnosis [6].

### 3.2. Imaging Features

Few imaging modalities are employed in the diagnosis of venous compression syndromes [8]. Contrast-enhanced chest CT and diagnostic venography are imaging modalities that can be utilized to diagnose AVC. Initial imaging with ultrasound may demonstrate sluggish flow and/or intraluminal thrombus in the left subclavian, brachiocephalic, or left IJV, however, these findings are nonspecific as they primarily relate to proximal stenosis or obstruction, and thus further investigation into AVC or brachiocephalic vein compression is warranted with cross-sectional imaging [7]. Based on a retrospective review of the literature, the importance of cross-sectional imaging for the evaluation of anatomical variation and in establishing the etiology of venous compression has been established [2, 8, 9]. Therefore, in the appropriate clinical setting, chest CT (with inspiratory and expiratory phase) can be utilized as the initial imaging modality to exclude an extrinsic venous compression etiology such as a mediastinal mass, aortic aneurysmal dilation, or compression secondary to the aortic arch anomaly. Even though the rare association of AVC with the aberrant right subclavian artery is noted in the literature, such a finding can be visualized on contrast-enhanced CT [7]. Once the brachiocephalic vein compression is suspected on CT, the next step of performing a diagnostic venogram can aid in real-time fluoroscopic evaluation, again in the setting of the inspiratory and expiratory phase, with the latter demonstrating venous compression. Secondary findings on the venogram, demonstrated by underlying collateral vascularity establish the chronicity of the diagnosis. The venogram can be performed via cannulating the basilic, cephalic or brachial veins, followed by advancing an angled catheter and a 0.035 Glidewire to access the distal left subclavian or axillary vein (proximal to the region of the stenosis). Alternatively, the catheter can also be advanced into the ipsilateral jugular vein. The angled catheter can be exchanged for a multipurpose side-hole catheter, through which a contrast injection can be performed in the inspiratory and expiratory phases. The patency within the left subclavian vein, LIJ, and left brachiocephalic vein during inspiration excludes an underlying acute or chronic thrombus as an etiology, thus highlighting the importance of a biphasic exam [2]. Therefore, the ability to visualize flow dynamics in venography can aid in establishing a firm AVC diagnosis, and thus venography could potentially be established as a gold standard [10, 11].

Moreover, during cross-sectional imaging, the AP diameter (dorsal aspect of the manubrium to the ventral aspect of the vertebral body) can be measured, at the level of the brachiocephalic vein in both inspiratory and expiratory phases of the examination. Upon expiration, the AP diameter diminishes, with a confined mediastinal anatomic space. Even though there are no established AP diameter parameters, a significant decrease in expiratory phase diameter should prompt further investigation into AVC or brachiocephalic vein compression. Furthermore, aberrant right subclavian arteries, which are the most common embryologic abnormality of the aortic arch, result as an additive feature to the already confined anatomic space in the mediastinum which further predisposes patients to brachiocephalic vein compression [2, 12]. Therefore, upon findings of an aberrant right subclavian artery with the appropriate abovementioned clinical symptoms, the patient could be assessed for AVC as a potential etiology once other differentials have been excluded. Nevertheless, standard variations in AP diameters on inspiration versus expiration, and the incidence of aberrant right subclavian arteries as the cause of AVC have yet to be established, drawing attention to the need for future studies on this topic.

## 4. Treatment and Discussion

Conservative management remains the mainstay in AVC and includes arm elevation at night as well as avoidance of the left lateral decubitus position to reduce pressure on the brachiocephalic vein [2]. In our institution, conservative management has proven to be sufficient in managing the symptoms and complaints of AVC. However, surgical management may be required for symptomatic cases of compression in the setting of conservative management failure. Yet, surgery is an invasive option that carries risks and thus is not recommended as a first-line approach. Therefore, conservative management is the advisable treatment option in symptomatic venous compression patients as a first-line approach. In patients who fail conservative management, angioplasty and self-expanding stents are treatment methods of choice in common venous compression syndromes such as Paget-Schroetter syndrome (PSS), nutcracker syndrome, and May–Thurner syndrome [13]. A literature review also demonstrated that self-expanding stents can successfully treat symptomatic stenosis of the brachiocephalic vein, particularly in hemodialysis patients where dialysis access may be threatened [4]. However, due to the extrinsic nature of compression, stents are not recommended in AVC patients due to limited patency and allowable volume changes of the vascular space during respiration [7, 14]. In the setting of the patient not being a surgical candidate and failing conservative management, the stenosis could potentially be treated with self-expandable stents. Notably, balloon expandable stents should also be avoided in AVC management as they can be crushed and can migrate secondary to external compression [7]. In severe cases of venous compression syndromes, operative decompression of the thoracic inlet including manubrioplasty has been employed as a radical approach to address the underlying anatomy [12]. Nevertheless, the long-term prognosis is unknown due to limited cases in the literature.

## 5. Conclusion

AVC is a rare extrinsic venous compression syndrome. Through the abovementioned case series, we highlight the importance of combining imaging modalities, particularly chest CT and venography, in confirming a diagnosis and determining the etiology of AVC.

## Data Availability

Data is available within the secure institutional electronic medical record in which the senior author is faculty.
